# Occult colon cancer with liver abscess and pancreatitis as the first manifestations: A case report

**DOI:** 10.1097/MD.0000000000032654

**Published:** 2023-01-20

**Authors:** Shan Yang, Jin Zhao, Qi Liu

**Affiliations:** a Department of Gastroenterology, Affiliated Hospital of Guizhou Medical University, Guiyang, People’s Republic of China.

**Keywords:** Colon cancer, *Klebsiella pneumoniae*, liver abscess, severe acute pancreatitis

## Abstract

*Klebsiella pneumoniae*-induced liver abscess (KP-PLA) is a common type of pyogenic liver abscess, severe acute pancreatitis (SAP) has high mortality, and poor prognosis in advanced colon cancer. There have been no report of SAP complicated with colon cancer after KP-PLA as so far. In this study, we reported a case of SAP secondary to KP-PLA with colon cancer for the first time, so as to provide reference for clinical diagnosis and treatment of these diseases.

**Patient concerns and diagnosis:**

A 64-year-old woman with a history of diabetes visited our hospital with abdominal pain for 5 + days. He was diagnosed with KP-PLA a month ago, which had not healed when he was admitted. He was diagnosed with SAP, and histological examination of colonic biopsy confirmed the diagnosis of moderately differentiated adenocarcinoma.

**Interventions and outcomes:**

He was treated with intravenous antibiotics and underwent modified endoscopic mucosal resection under colonoscopy. We conducted a 2-month follow-up, and there was no recurrence of liver abscess and pancreatitis.

**Conclusion:**

Screening for intestinal tumors is necessary in patients with cryptogenic liver abscess, especially KP-PLA with diabetes.

## 1. Introduction

Pyogenic liver abscess (PLA) is a serious infectious disease and *Klebsiella pneumoniae* is the most common bacteria in PLA in our country.^[1]^ Endophthalmitis caused by *Klebsiella pneumoniae* induced liver abscess (KP-PLA) was first reported in Taiwan in the 1980s,^[2]^ and some rare complications of liver abscess such as brain abscess and lung abscess have been reported successively in recent years.^[3,4]^ As medicine progressed, acute pancreatitis (AP) was characterized by bacterial infections, including salmonella and tuberculosis, in addition to the common causes such as Biliary tract disease, alcohol, and high triglycerides.^[5,6]^ *Klebsiella pneumoniae* usually causes secondary infection after AP, there have been no cases of primary infectious pancreatitis caused by *Klebsiella pneumoniae* excluding other causes. The fatality rate of severe acute pancreatitis (SAP) is as high as 40%,^[7]^ and the 5-year survival rate of advanced colon cancer is significantly lower than that of early colon cancer.^[8]^ Therefore, it is particularly important to avoid secondary SAP of KP-PLA, and early diagnosis and treatment of colon cancer. At present, there has been no report of KP-PLA secondary SAP complicated with colon cancer at home and abroad. We report the first case of early colon cancer with KP-PLA and its secondary SAP as the initial symptom, aiming to provide reference for clinical diagnosis and treatment of this disease.

## 2. Case report

**Figure 1.**
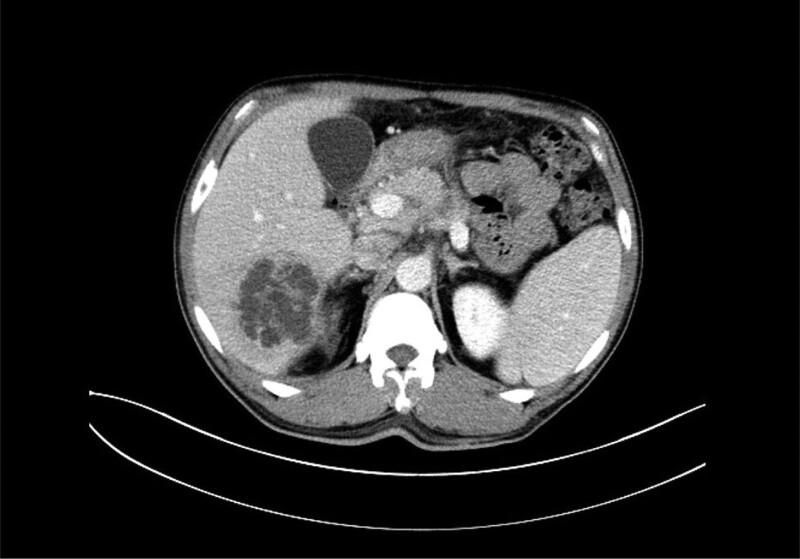
Abscess formed in S6 segment of liver.

**Figure 2.**
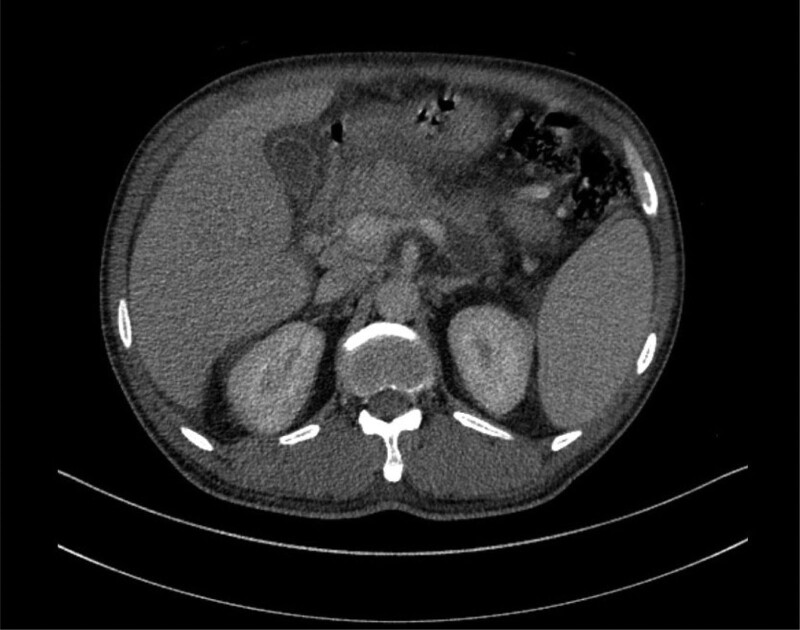
Acute pancreatitis with peripancreatic exudation and pancreatic cyst formation.

**Figure 3.**
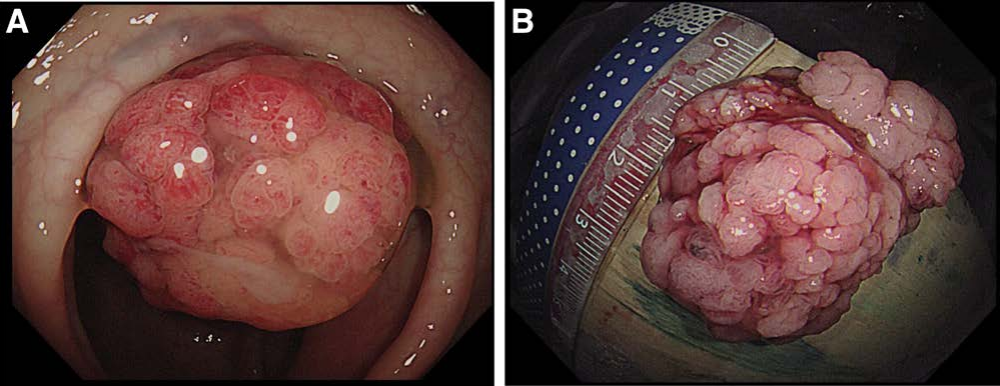
(A) Electronic colonoscopy of sigmoid mass, appearance of vegetable; (B) The measured size of the mass was about 3.5cm × 2.5 cm × 1.7 cm.

**Figure 4.**
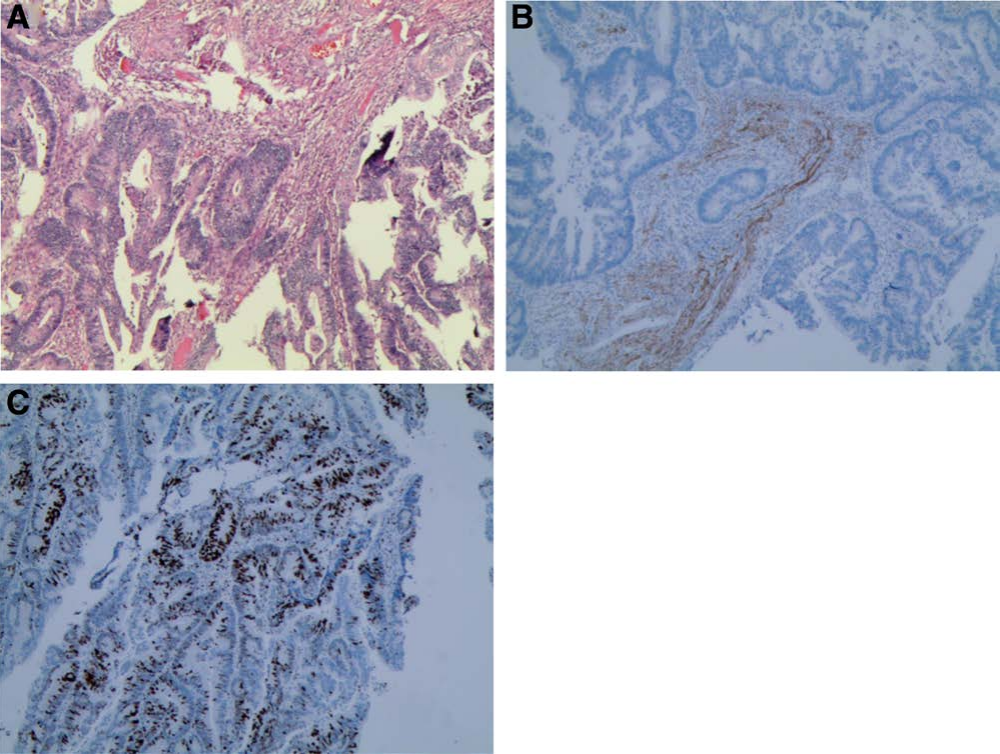
(A) The tumors were glandular in arrangement under microscope, and some cells had atypia (HE, ×40). (B) Desmin staining +. (C) Ki-67 < 30%.

**Figure 5.**
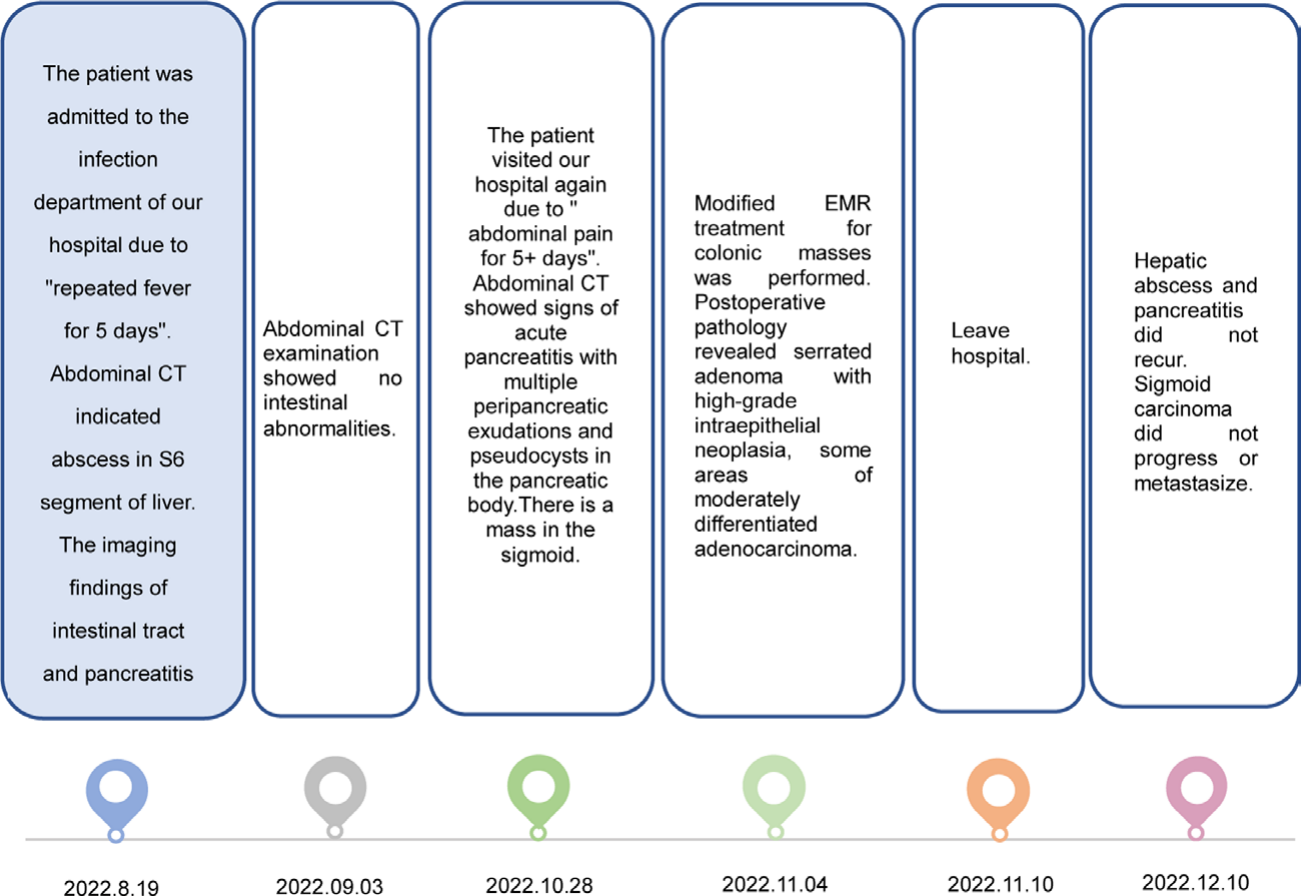
The whole diagnosis, treatment, and prognosis of this case.

The 51-year-old male patient was admitted to the infection department of our hospital on August 19, 2022 due to “repeated fever for 5 days.” He has a history of “type 2 diabetes,” irregular oral treatment with “metformin,” and unknown fluctuations in blood sugar levels. Physical examination on admission: temperature 38.3°C, tachycardia and otherwise normal vital signs. Laboratory tests showed increased neutrophil percentage (NEUT%), C-reactive protein, and procalcitonin. Hemoglobin A1c (HbA1c) 13.01%. *Klebsiella pneumoniae* was cultured in both the drainage fluid and venous blood. Abdominal CT scan indicated abscess in S6 segment of liver (Fig. 1). The imaging findings of intestinal tract and pancreatitis were normal. Piperacillin/tazobactam were used, and percutaneous hepatic abscess puncture drainage was performed. During hospitalization, the patient’s blood glucose fluctuated between 4.2 and 27.7 mmol/L. He refused the insulin hypoglycemic program and only gave temporary insulin for blood glucose control 1 + month later, the patient visited our hospital again due to “ abdominal pain for 5 + days.” Vital signs were stable, lung, and heart physical examination was not special; Abdominal tenderness, no rebound pain and muscle tension, bowel sound about 1 time per min. Laboratory tests showed that NEUT% was 85.6%, C-reactive protein 164.82mg/L, and procalcitonin 3.66 ng/mL. Abdominal CT scan showed signs of acute pancreatitis with multiple peripancreatic exudations and pseudocysts in the pancreatic body (Fig. 2).There is a mass in the sigmoid. He was diagnosed with SAP. After admission, the patient developed fever and was treated with piperacillin/tazobactam for 3 days. The patient’s body temperature was still repeated, and after the antibiotics upgraded to meropenem, his body temperature gradually returned to normal. During treatment, his fluctuation of blood glucose was measured at 6.7 to 23.9mmol/L. Regular insulin treatment was refused and insulin was given for temporary hypoglycemia. Then his abdominal pain eased and his inflammatory markers went down. After the disease improved, colonoscopic modified endoscopic mucosal resection treatment for colonic masses was performed (Fig. 3). Postoperative pathology revealed serrated adenoma with high-grade intraepithelial neoplasia, some areas of moderately differentiated adenocarcinoma (Fig. 4). Hepatic abscess and pancreatitis did not recur after 2 month of follow-up. The whole diagnosis, treatment, and prognosis of this case as shown in Fig. 5.

## 3. Discussion

KP-PLA can cause extrahepatic metastatic infection, but its secondary SAP has not been reported so far. The patient had no history of biliary tract or pancreatic diseases and drugs taken that may cause pancreatitis, no excessive drinking or overeating before the onset, normal triglyceride level, and negative autoimmune related antibodies. And he have had KP-PLA before the occurrence of pancreatitis, so KP-PLA secondary SAP was considered. By reviewing relevant literature, the potential mechanism of secondary AP of KP-PLA is proposed here: blood infection: *Klebsiella pneumoniae* was cultured in the drainage fluid and blood of some KP-PLA patients with secondary extrahepatic infection, suggesting that *Klebsiella pneumoniae* could cause extrahepatic infection through blood transfer.^[3,9]^ A retrospective study suggested that KP-PLA patients with sepsis were more prone to extrahepatic infection, suggesting that hematogenous infection was a viable route.^[10]^ Biliary tract infection: According to the anatomical structure of liver and pancreas, the intrahepatic and intrahepatic bile ducts eventually converge to the common bile duct and accompany the pancreatic duct to eventually open at the duodenal papilla; part of the common bile duct may converge with the pancreatic duct and open at the duodenal papilla; bacterial infection in the liver may enter the pancreatic tissue through the biliary tract system and lead to AP. Hepatopancreatic fistula and pancreatic portal vein fistula: There have been cases reported in the past that secondary hepatic abscess in patients with chronic pancreatitis is due to the formation of hepatopancreatic fistula,^[11]^ and the formation of pancreatic portal vein fistula can lead to multiple focal hepatic abscess secondary to acute pancreatitis.^[12]^ If hepatic abscess rupture causes hepatopancreatic fistula, bacteria can also infect pancreatic tissue through fistulas and lead to AP. Patients with diabetes are more likely to suffer from *Klebsiella pneumoniae* infection, and KP-PLA patients with high blood glucose level are more likely to develop extrahepatic metastatic infections.^[13,14]^ The generally accepted theory is that high glucose levels reduce the adhesion, chemotaxis, phagocytosis of neutrophils, and increase the aggressiveness of Klebsiella pneumoniae.^[15]^ The most common bacterial infection of PLA originates from the biliary tract, and some cryptogenic liver abscesses with unknown infection source were reclassified as enteric after intestinal examination, even were associated with intestinal tumors.^[16]^ Unfortunately, the patient had no intestinal infection symptom when he was admitted to hospital for the first time due to liver abscess, which was classified as cryptogenic liver abscess without complete colonoscopy, thus missing the opportunity for early screening of intestinal tumors. He was diagnosed with colon cancer due to SAP readmitted to hospital, suggesting that liver abscess and extrahepatic metastatic infection were closely related to delayed diagnosis of colon cancer. Colon cancer is one of the cancers with the highest prevalence in the world, its early clinical symptoms are not typical, and most of them are found in the late stage, so the mortality is high.^[17]^ Although there are no guidelines to determine whether PLA patients should be routinely screened for colon cancer, many scholars believe that PLA is closely associated with colon cancer, especially in patients with *Klebsiella pneumoniae* infection and diabetes.^[18,19]^ Mohan et al conducted a meta-analysis, which showed that patients with PLA had a much higher incidence of colon cancer than the general population, and over 90% of the pathogenic bacteria were Klebsiella pneumoniae. Therefore, early colon cancer screening for patients with cryptogenic KP-PLA was recommended.^[20]^ It is generally accepted that colon cancer leads to the destruction of intestinal mucosal barrier, and enteric bacteria enter the blood along with the portal vein to cause liver abscess.^[21]^ In addition, the relationship between diabetes and colon cancer is also attracting increasing attention. A large cross-sectional study showed that diabetes mellitus significantly increases the risk of gastrointestinal tumors, especially colorectal cancer.^[22]^ It has been reported that hyperinsulinemia promotes the proliferation of tumor cells by stimulating the IP3-kinase/AKT pathway,^[23]^ apoptosis was inhibited by up-regulation of insulin-like growth factor-2,^[24]^ hyperinsulinemia promote the development of colon cancer by stimulating mutations in the DNA bases of colon cells.^[25]^ A case of brain abscess due to delayed diagnosis of KP-PLA associated with colon cancer was reported in Japan in 2000.^[26]^ This case is the first time KP-PLA associated with colon cancer has been proposed to cause SAP.

## 4. Conclusion

KP-PLA is closely associated with colon cancer. Even in the absence of intestinal symptoms and positive radiographic findings, screening for intestinal tumors in cryptogenic liver abscesses is necessary, especially in patients with *Klebsiella pneumoniae* infection and diabetes mellitus. Early removal of the etiology can not only reduce the recurrence of PLA, but also reduce the probability of complications of extrahepatic infection. As a rare complication of KP-PLA, the pathogenesis is not very clear, but blood infection is a feasible route, so the treatment of sepsis is helpful to shorten the treatment time and improve the prognosis. High blood glucose level can promote the extension of infection, stabilizing blood glucose level is an important measure to reduce extrahepatic metastatic infection in KP-PLA patients.

## Author contributions

**Data curation and investigation:** Shan Yang.

**Supervision:** Qi Liu.

**Writing—original draft:** Shan Yang.

**Writing—review and editing:** Jin Zhao.
